# Book Review: The Neuroscience of Creativity

**DOI:** 10.3389/fpsyg.2021.643236

**Published:** 2021-02-18

**Authors:** Aspasia Eleni Paltoglou

**Affiliations:** Department of Psychology, Manchester Metropolitan University, Manchester, United Kingdom

**Keywords:** creativity, cognitive neuroscience, scientific creativity, experiments, limitations

What is creativity? How can we quantify it and measure it reliably? What happens in our brains when we create a poem, a story, a symphony? Anna Abraham's book “The Neuroscience of Creativity” includes a fascinating discussion of neuroscientific research on creativity as well as relevant theories.

Although at first glance creativity could be perceived as something difficult to pin down, something best left to poets and humanities scholars, Abraham convincingly shows that it can be successfully operationalized and examined neuroscientifically. As she points out, the two main components of creativity are originality and appropriateness within a context.

The author adopts Rhodes' approach (1961) of investigating creativity from the point of view of process, person, place and product, which is a very useful framework when thinking of creativity in scientific terms. In other words, one can explore creativity from one or more of these four different perspectives: by examining (1) the creative product; (2) the personality characteristics and habits of the person coming up with original and appropriate ideas and products; (3) the extent to which the environment is conducive to creativity; (4) and, last but not least, the internal mental processes (such as perception, learning) involved in creativity. The most prevalent approach from a neuroscientific perspective is the last approach.

Having armed the reader with essential knowledge on what creativity is and how it can be studied, Abraham discusses creativity within the framework of creative cognition. The author describes several different cognitive models that attempt to explain the creativity process, such as the defocused attention and disinhibition models.

Abraham then turns to the brain. Which parts of the brain are involved in creativity? Is it even possible to relate such a complex process to certain brain areas? The author discusses studies that focus on individual brain areas, as well as those that would rather examine brain networks, such as the Default Mode Network and the Central Executive Network. There is an excellent critical account of dual models and associated brain networks, which permeate cognitive neuroscience. Typically, textbooks tend to describe this dual framework, without highlighting the underlying assumptions and limitations. It was therefore refreshing to see a critical account of this influential and ubiquitous framework.

This is just one example of the frank and in-depth critical evaluation of the relevant research and theories contained in this book. Abraham does not just describe the relevant studies and theories, but trains the reader to see through the assumptions and limitations thereof. This is particularly the case in Chapter 5, where the challenges of neuroscientific research on creativity are discussed. So can we study creativity (neuro)scientifically? Yes, although conducting rigorous experiments is far from easy. Abraham lays bare the problems that plague this research area, such as subjectivity when judging creative products, and the non-binary nature of responses of the tasks employed to measure creativity. The author calls for theory-driven experiments that are ecologically and biologically valid with suitable controlled conditions.

I have experienced first-hand the difficulties that can arise when conducting cognitive neuroscience experiments, issues that do not tend to be discussed so explicitly in similar textbooks. Interestingly, Abraham mentions that it is extremely difficult to investigate certain types of creativity in the neuroscientific laboratory, such as spontaneous creativity. It is certainly wise to admit that certain types of behavior are almost impossible to explore in the laboratory, and instead focus efforts on more feasible endeavors.

Nevertheless, looking to the future, I cannot help but wonder: will areas that are challenging to research presently, remain forever out of reach? Given the technological advances and spread of the use of physiological tools such as smart wearable health devices and phone apps, I wonder whether neuroscience equipment might soon benefit from similar advances. That would enable brain activation to be recorded as participants go about everyday life, and perhaps enable rigorous studies on challenging subjects such as spontaneous creativity.

The book includes a brief set of recommendations for improved research practice, including authors being open about strengths and limitations of studies on creativity, and using multiple techniques to investigate a research question. Perhaps a more detailed account of problems and improvement recommendations in creativity research could have been included as a comprehensive guide for future researchers. Personal accounts of experts on neuroscience of creativity would have also highlighted the issues further.

Ironically, it turns out that scientific creativity is under-investigated compared to artistic creativity. One area for investigation that I think would be of interest is the effect of the extensive training on scientists' creative ability to come up with new theories, studies and methods. The importance of being able to come up with creative scientific solutions has been brought to the fore, as humanity is looking for solutions to a very troubling pandemic. In fact, I wonder if the author could have reflected on her own research journey in relation to neuroscience of creativity, and included examples of moments of insight, frustration, and creative problem solving, and link them back to empirical studies and theories discussed. It would have also been fascinating to know a little more about how she became interested in the neuroscience of creativity. Reflection is less common in quantitative than qualitative studies, but I think it is very useful and illuminating in any area of research.

Nevertheless, this is an excellent textbook. Abraham provides a frank and clear overview of the research and challenges in this fascinating research area. The book is written in a clear and compelling manner and the author's passion about the subject shines through. Abraham states that this is the book she would have liked to have had when she started her research adventure. I think anyone interested in creativity would be grateful for this book.

## Author Contributions

The author confirms being the sole contributor of this work and has approved it for publication.

## Conflict of Interest

The author declares that the research was conducted in the absence of any commercial or financial relationships that could be construed as a potential conflict of interest.

